# Memory on the move

**DOI:** 10.1007/s00018-012-0964-y

**Published:** 2012-04-06

**Authors:** Andreas Radbruch

**Affiliations:** Deutsches German Rheumatism Research Centre (DRFZ) Berlin, an institute of the Leibniz Association, Berlin, Germany

**Keywords:** Stromal Cell, Plasma Cell, Helper Cell, Antigenic Challenge, Immunological Memory

Immunological memory, next to specificity and tolerance, can be regarded as one of the key characteristics of the adaptive immune system, adapting it to the antigenic challenges and pathogens of its environment. Knowledge about the acquisition of “Immunity” to recurrent pathogens is cultural heritage of mankind. The Greek writer Thucydides in 400 b.c. mentions it in his chronicles as established knowledge [1]. This knowledge has also been exploited early on, “inoculating” children with attenuated pathogens, a custom that reached Europe from Asia, as discussed by Voltaire in 1733 [2], and later marketed as “vaccination” according to Jenner in 1798 [3].

When Behring and Kitasato demonstrated the specific protection of rabbits by serum from infected animals in 1890 [4], it became clear that serum antibodies could provide “immunity”, i.e., protection against a recurrent infectious challenge. It took another 60 years until Astrid Fagreus discovered the cells secreting those antibodies, the “plasma cells”, describing them as “terminal stages of B cell differentiation” [5]. “Protective memory”, as provided by serum antibodies, was later conceptually complemented by a “reactive memory” formed by expanded and long-lived populations of antigen-experienced T and B lymphocytes.

The nature of this reactive memory and its role in “immunity” is a matter of debate even today. The nature of “protective memory” became less controversial recently, since it has been shown in the past 15 years that plasma cells can survive as “memory” plasma cells in the bone marrow, secondary lymphoid organs and inflamed tissues, in dedicated “survival niches” organized by specialized stromal cells [6–9]. This concept, challenging the traditional view of long-lived effector cells and/or memory cells with a “halflife” and “homeostatic” regeneration of memory, emerged when analyzing the lifestyle of long-lived plasma cells. Could it be a general principle of the organization of memory? Tracking T helper cells through the immune reaction, to protein antigens mimicking vaccines, into the “memory phase” surprisingly showed that while the cells gradually disappeared from the periphery, about 20 % of them were stably maintained alive in the bone marrow in niches similar to those of the plasma cells but definitely organized by different stromal cells [10]. The T helper cells from bone marrow were resting in terms of transcription and proliferation, but highly reactive to restimulation and potent helpers of activated B cells. These cells pose an immense challenge to the traditional views on “reactive memory”. Antigen-experienced T helper, cytotoxic T cells and B cells are present in the periphery, they can be long-lived, they are functionally diverse, and they can provide protection upon adoptive transfer. What is their role as compared to the resting bone marrow cells? How are the T cells of bone marrow reactivated upon antigenic challenge? Are cytotoxic T cells and B cells also resting in the bone marrow? In short: Analysis of immunological memory is on the move! It is at this break that we here assemble the viewpoints of eminent scientists who have contributed key concepts to our current views an immunological memory.

In the first essay, Zehn and colleagues discuss how the original activation of T cells by antigen impacts their activation and development into long-lived cells. Yamane and Paul then provide a look into the T cells themselves, in particular their functional polarization and imprinting, and their developmental plasticity. This is also discussed by Buchholz and colleagues, for CD8+ T cells, in particular with respect to the question whether effector and memory cells are distinct lineages or not?

Boyman and colleagues then discuss the maintenance of long-lived T cell and NK cell populations of the periphery by “homeostatic” proliferation, in the apparent absence of antigen. The recently identified memory T helper cells from bone marrow, described by Tokoyoda and Radbruch, are resting in terms of proliferation. While we poorly understand their relation to peripheral effector memory cells, they provide a new model of organization and maintenance of memory by stromal cells. This may be a general principle of organization of memory, since it has recently been shown that also long-lived memory plasma cells are organized by bone marrow stromal cells. In a final view on T cell memory, Goronzy and Weyand draw our attention to the aging memory and its potential for a break of tolerance and induction of autoimmune diseases.

The discourse is closed by two provocative essays. Reynaud and colleagues discuss B cell memory, in particular the controversial facet of IgM memory B cells, which are a remarkable population in humans, and not so in mice. This raises the general question of how to model long-lasting memory in short-lived mice. Not to forget the memento of Rolf Zinkernagel, to put memory in its biological context, and to understand it with respect to its protective value, which brings us back to Thucydides.
